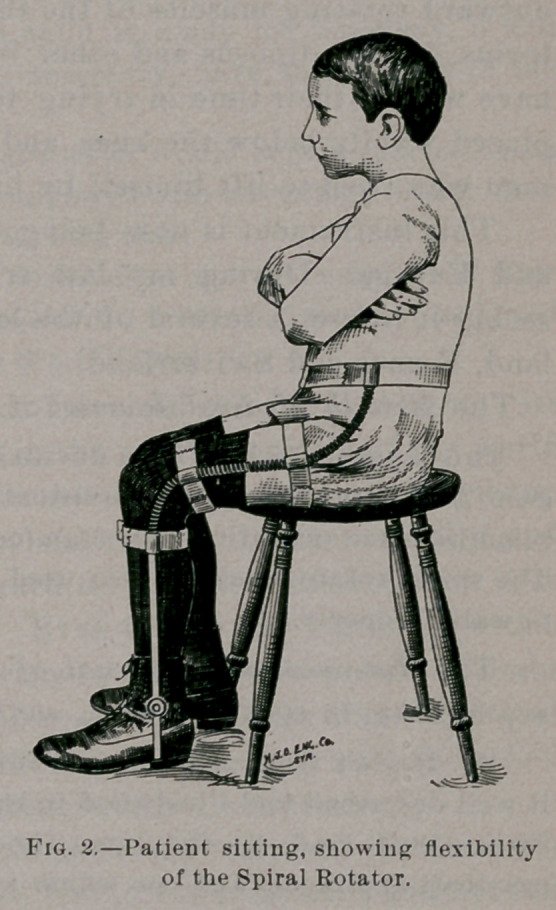# Some New Features in the Treatment of Club-Foot

**Published:** 1895-07

**Authors:** Gregory Doyle

**Affiliations:** 307 West Genesee Street; Syracuse, N. Y.


Buffalo Medical and Surgical Journal

VOL. XXXIV. JULY, 1895. No. 12.

### Original Communications.

### SOME NEW FEATURES IN THE TREATMENT OF CLUB-FOOT.

BY GREGORY DOYLE, M. D., Syracuse, N. Y.

THERE is no deformity of the human body more distressing than that of club-foot, and none which has more severely taxed the ingenuity of orthopedic surgeons.

The various modes of treatment and the many different appliances devised for its correction are nearly as numerous as the investigators of this interesting subject.

The etiology of congenital talipes is also a fruitful topic for discussion, but it is very evident to my mind when all the arguments are summed up that it is due to uterine pressure, and that only.

The treatment for correction cannot be commenced too early. The nurse should be instructed to turn and hold the deformed feet of the new-born babe in as nearly a normal position as possible as often as convenient, and to continue this practice, alternated with proper massage, until such time as the surgeon may deem it proper to operate.

This manipulation a is great adjuvant to subsequent surgical treatment when such is found necessary, as it tends to develop the weakened muscles and to bring into prominence the contracted tendons. In some very mild cases the deformity may be remedied by properly directed traction with adhesive straps. Cases that can be completely corrected by this means are, however, of the very mildest type.

Where the deformity is great and the contraction firm, nothing short of subcutaneous tenotomy will suffice. Many attempts have been made to correct this malformation by forcibly stretching the firmly contracted tendons and muscles with powerful instruments.

Instead of benefit accruing from this procedure much injury has been done, and cases that were amenable to proper treatment were thus rendered permanently incurable.

Subcutaneous tenotomy, when properly performed, is a perfectly safe operation, and will accomplish better results than any other manner of treatment. There are some tendons, however, which will be found difficult to completely divide subcutaneously without wounding adjacent vessels and nerves. In such cases it is far better to make the open operation. This may be done by opening down to the tendon, parallel with it, and dissecting it away from all attachments. It is then to be drawn upward from its normal position with a blunt tenaculum and completely divided. I have operated in this way many times and find it very satisfactory. Of course, complete asepsis must be maintained, otherwise the result may be unfortunate, as suppuration might develop and thwart the object of the operation.

After tenotomy I immediately place the foot in a normal position and retain it there with adhesive straps applied in such a manner as to hold it firmly. Some operators object to this procedure and claim that the foot should be left in its abnormal position for a few days after the operation. Their reason for this, no doubt, is that the space left by the retracted ends of the divided tendons will be filled with blood if farther drawn apart, which, they imagine, might interfere with reunion. We know that when the tendon of a contracted muscle is completely divided there will be an immediate separation of the ends, even when the foot is not held in a normal position. This precaution is, therefore, shown to be unnecessary.

It has been my practice to unhesitatingly restore the foot to its normal position immediately after the operation, and I have been indorsed in this procedure by no less an authority than Dr. Lewis A. Sayre, of New York. The space between the tendons, no doubt, will fill with blood, but when the operation is done with perfect asepsis there is no danger of suppuration, and the blood remaining in the cavity goes to make up new material for splicing out the tendon. I have always operated in this way and have every reason to be perfectly satisfied. The best arguments in any discussion are solid facts. I have never seen any failure of union of the tendons when correction, and even over-correction, of the foot has been immediately made; my opportunities for observation in my own cases and those of others have been abundant during the past thirty years, and I may safely say that I have never heard of a failure where the operation was skilfully performed. Nothing is gained by leaving the foot in its abnormal position for a few days after tenotomy ; it only endangers the necessity and enhances the difficulties of a second operation.

As to the different appliances used to retain the foot in its normal position, many are good, some are indifferent and a host are worse than useless. Of course, different appliances must be used for the various forms of talipes. At present I shall deal only with talipes *equino varus*, the most frequent form of club-foot. To retain the normal position of the foot after thorough tenotomy,

FIG. 1.—Patient walking, showing the feet everted by Spiral Rotator.

FIG. 2.—Patient sitting, showing flexibility of the Spiral Rotator.

plaster of Paris has been used, but it has not proved a success, as it is liable to break and crumble ; it is heavy, clumsy and apt to soften. Proper application of adhesive straps after tenotomy proves very successful and many of my cases have made complete recovery with this simple dressing alone.

Even after successful tenotomy and correction there still exists a strong tendency to inversion of the feet, which is almost as unsightly a deformity as the original trouble, and tends to produce a relapse. To remedy this obstinate difficulty I devised, in the summer of 1880, an appliance which I term the spiral rotator. It is made of flexible spiral spring shafts, attached to a pelvic belt at their upper ends and to the soles of the shoes at the lower ends. When this is properly fitted and applied to the patient, his feet and limbs can be rotated to any desired angle. While wearing it the patient is able to assume any desired position without interrupting the automatic action of the instrument.

It seems to be overlooked by many surgeons that in a great majority of cases of talipes *equino varus* the entire limb is rotated inward, rotation taking place in the acetabulum. This is the result of partial paralysis of the gluteus maximus and quadratus femoris—outward rotating muscles of the thigh—and contraction of the sartorius, semitendinosus and other inward rotating muscles. Many have wasted their time in trying to effect eversion with appliances placed wholly below the knee, and have had as much success as the man who tried to lift himself by his boot straps.

This instrument is now being extensively used in this country and Europe. During my last trip abroad I had the pleasure of seeing it in use in several of the leading hospitals in England, Ireland, France and Switzerland.

The *British Medical Journal* of February 12, 1881, says:

> This apparatus has been devised by Dr. Gregory Doyle, of Syracuse, N. Y., to remedy the persistent inversion of the foot, which often remains after operations on club-foot, especially talipes *equino varus*. The spiral rotator may be also used in educating pigeon-toed children to walk properly.

The *International Journal of Medicine and Surgery*, of January 8, 1881, in speaking of it, says:

> Dr. Doyle's appliance already enjoys great popularity, and we find it well described and illustrated in the *Polytechnich Journal* of Berne, Switzerland, and, at the same time, highly recommended as a very practical appliance, and one which will soon be in universal demand.

The Philadelphia *Medical Times* of December 18, 1880, says:

> In applying this apparatus the pelvic and thigh bands are secured, when, if the case be one of varus, the shoe is to be rotated once inward, the foot is then placed in it and fastened by lacing. It will at once be seen that the constant tendency of the spring will be to uncoil itself into its original position, and in doing this it must carry the toes outward. In valgus the action would be reversed. It is a force exerted gently, constantly and coaxingly, awake and asleep, and, if increased power be required, the shoe can be rotated twice or three times before it is applied to the foot.

A great advantage obtained by its use is the fact that it in no wise interferes with either the motion of the leg or the action of any muscle, save the one that it is intended to antagonise. Its tendency to straighten itself as a whole would, in a slight degree, raise the anterior part of the foot, and to that extent beneflt even an equino. As I have just said, it does not act with much force upon the medio-tarsal joints, yet its applicability to very young children is great in its favor, as it can thus be used as a continual assistant to manipulation. As the hands of the surgeon cannot always be in service, and as the mother will not do this more than fifteen minutes during each day, it will be seen that a force acting steadily throughout the remaining twenty-three and three-quarter hours will prove no mean accessory. Such a splint applied even during the first week of a child's life could do no harm. By the time that three months have passed, and the child is ready for tenotomy, it will be found that no operation will be necessary, save the section of the tendo-achillis.

For pigeon-toed cases this spiral rotator would be most perfect in its action. and the principle could be employed with advantage on any limb which is deformed by rotation on its long axis.

The inventor has used it for torticollis, and also employs it after division of tendons in varus and equinus. I like the mode of action of the spiral and shall certainly give it a full trial.

It is now fourteen years since I devised the spiral rotator. Its uniform success and popularity in the great hospitals of the world has given it a permanent place in the armamentarium chirurgicum. It is the only instrument yet devised that will automatically and successfully rotate the leg outward or inward, while the limb is in an extended or flexed position. Even to this day I hear of orthopedic surgeons seeking some means to rotate their patient's limbs after operation on talipes. If they will consult the transactions of the American Medical Association for 1880, they will there find a full description of it, supplemented with illustrations.

The reason many club-feet are not completely and permanently corrected is that the people often tire of the treatment and become impatient and neglectful in following out the surgeon's instructions. They must remember that there is no "time table" to go by in these cases, and also that the old proverb, "The way the twig is bent the tree's inclined," is very appropriate in the treatment of deformities. If the feet are kept in their normal position sufficiently long for nature to rebuild the deficient muscles and joints, satisfactory results will be obtained.

For the benefit of orthopedic surgeons I would state that Messrs. George Tiemann & Co., 107 Park Row, New York, make the spiral rotator, and they can be had with the spiral extending from the hip to the foot or to the garter only. Many prefer the latter for appearance sake, but the former is more flexible and allows greater freedom of motion.

307 WEST GENESEE STREET.

## Figures and Tables

**Fig. 1. f1:**
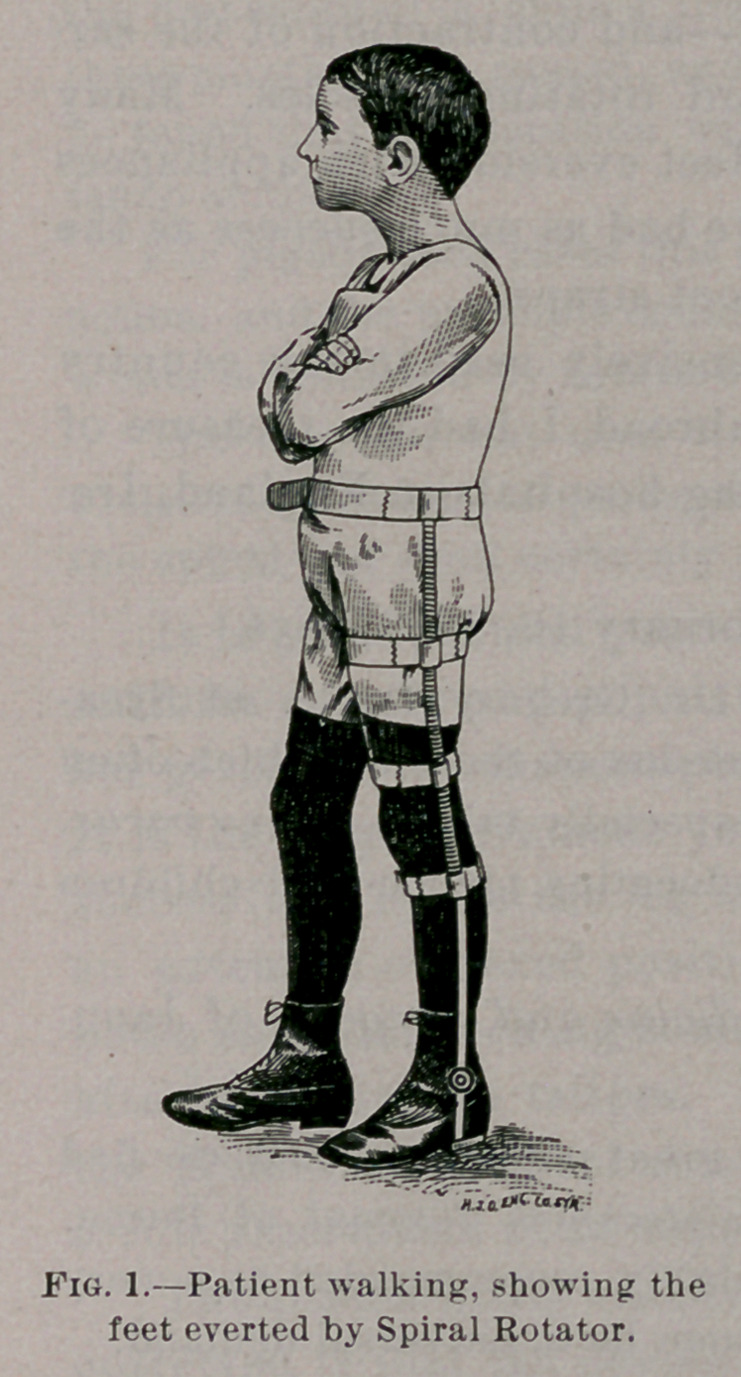


**Fig. 2. f2:**